# Senescence and extracellular vesicles: novel partners in vascular amyloidosis

**DOI:** 10.18632/aging.204571

**Published:** 2023-03-01

**Authors:** Meredith Whitehead, Marco Antonazzi, Catherine M. Shanahan

**Affiliations:** 1 British Heart Foundation Centre of Research Excellence, School of Cardiovascular and Metabolic Medicine and Sciences, King’s College London, UK

**Keywords:** amyloid, smooth muscle cells, senescence, extracellular vesicles, medin

Amyloidosis is a prevalent age-associated pathology caused by the accumulation of fibrous, insoluble protein fibrils in tissues. The most common human amyloid is aortic medial amyloid (AMA), caused by aggregation of a 50-amino acid peptide called medin, which is cleaved by an unknown mechanism from its parent protein, milk fat globulin EGF-factor 8 (MFGE8). Medin is present in the vessel wall of 97% of Caucasians aged over 50-years, yet despite its prevalence in the ageing population there is a very limited understanding of the mechanisms driving AMA. The novel data presented in the paper by Whitehead et al. provides evidence that vascular smooth muscle cell (VSMC)-derived small extracellular vesicles (sEVs) are key mediators of medin accumulation in the vessel wall [1]. In addition, the authors identify, for the first time, a role for cellular senescence in triggering amyloidosis via changes in sEVs and extracellular matrix (ECM) composition. Thus, this study not only advances our understanding of how AMA is formed but uncovers potential therapeutic targets for mitigating the detrimental effects of amyloidosis on tissue function.

Despite several forms of amyloidosis, including AMA and Alzheimer’s disease (AD), being frequently associated with ageing, there has been limited research to date on the effect of cellular ‘ageing’, termed senescence, on amyloidosis. Senescence is considered a stable cell cycle arrest provoked by various stresses associated with ageing, including persistent DNA damage and telomere shortening [2]. Senescent cells accumulate in the vasculature with age and undergo a range of phenotypic changes, including ECM remodelling and increased secretion of inflammatory mediators. Whitehead et al. confirmed a strong correlation between age and medin in the ECM of the medial layer of human aortic tissue. *In vitro* studies, using human VSMCs, demonstrated that the ECM synthesised by senescent cells contained medin in a fibril-like form. This was in contrast to the small, round aggregates found in the ECM from healthy, proliferative VSMCs, suggesting senescent cells create an environment permissive for medin aggregation. Further mechanistic studies found that sEVs secreted from VSMCs carried medin as a cargo and were responsible for medin release and also its aggregation in the ECM. Importantly, senescent VSMCs showed enhanced sEV secretion and senescent sEVs could accelerate medin aggregation compared with sEVs from proliferative VSMCs. The authors hypothesized that this was due to the formation of fibril-like amyloid seeds which were observed exclusively on the surface of senescent sEVs using super-resolution microscopy. To evaluate what factors might mediate these changes, proteomic analysis was performed on both sEVs and the ECM from senescent VSMCs. This demonstrated significant changes in sEV cargo-loading with senescence and also enrichment of the senescent ECM with the proteoglycan, HSPG2. Importantly, HSPG2 was shown to have a strong affinity for binding sEVs and sEVs from senescent VSMCs showed enhanced binding to HSPG2. The authors concluded that the changes in sEVs and the ECM during VSMC senescence synergise to trigger medin accumulation and aggregation into fibril structures in the aged ECM.

**Figure 1:**
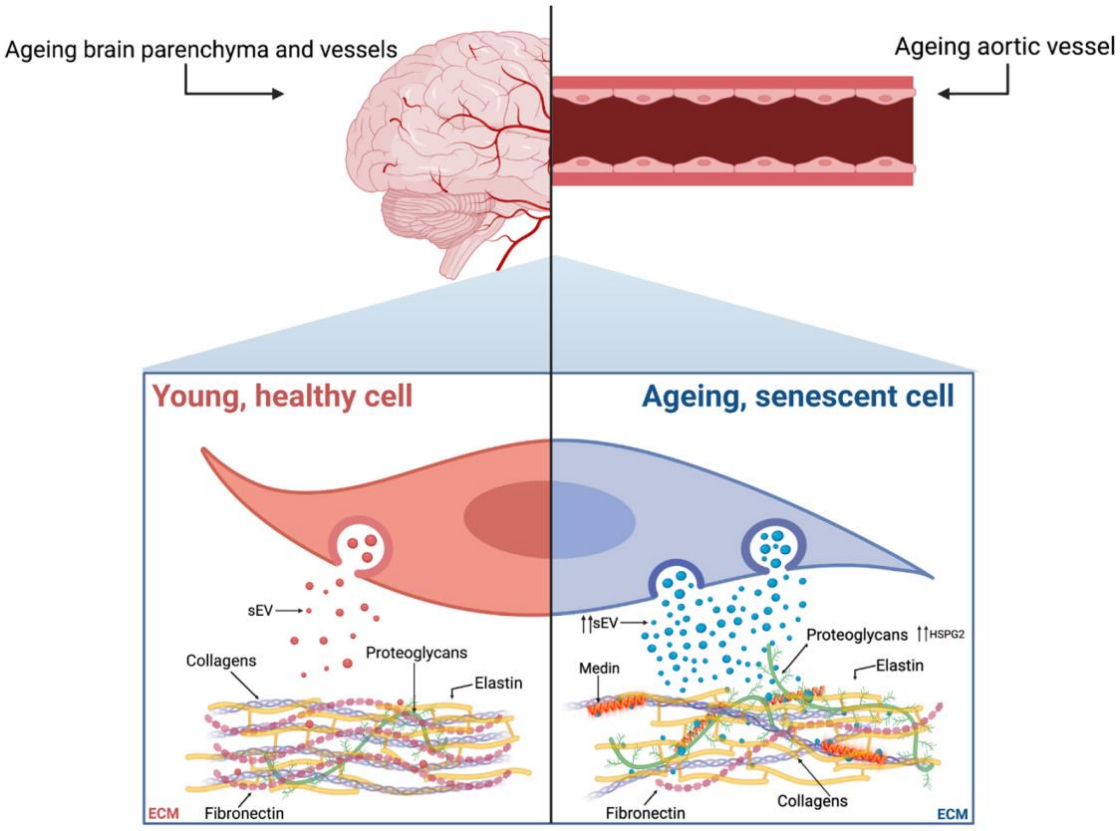
**Proposed mechanism showing the parallels between age-associated brain and vascular amyloidosis, with both being mediated by sEVs and affected by cellular senescence.** In the aorta, VSMC senescence acts as a trigger for medin accumulation, by increasing sEV secretion and enriching the ECM with HSPG2. These processes share similarities with the mechanisms regulating Aβ accumulation during AD and CAA, which could be key to understanding the build-up of medin in the brain and its colocalisation with other amyloid proteins. Created with https://BioRender.com.

However, there are a number of questions that remain unanswered. The physical role of sEVs on medin aggregation was not fully investigated and it would be interesting to understand what changes in senescence-associated sEVs trigger accelerated medin aggregation. This could be related to cargo-loading, although no clear candidates were identified by proteomics. Alternatively, the lipid composition of vesicles may be involved. Recent research has shown there are changes in lipid membrane composition in sEVs derived from senescent cells and lipid membranes have been shown to be important in amyloid fibrillation.

Interestingly, this study highlighted the many mechanistic parallels between vascular and brain amyloidosis. For example, it is well established that amyloid-β (Aβ), the amyloid associated with AD, can be secreted by small sEVs in the brain, which initiates its build-up in the extracellular space [3]. Several studies have also demonstrated an association between senescent neural cells, such as astrocytes, microglia and neurons in humans and mouse models of AD [4]. There has been some debate about the causal effects of cellular senescence on amyloidosis in the brain, but recent studies highlight a potential cause and effect relationship, with cellular senescence contributing to Aβ build-up, while Aβ itself can trigger senescence of neural cells [4]. It remains unclear whether there is a direct effect of medin on VSMC senescence and if there could also be a feedback cycle enhancing its accumulation and vascular dysfunction with age. These shared mechanisms have also provided insights into promising areas for intervention. Senolytics have been found to be effective in improving the damaging effects of Aβ brain plaques in mouse models [5], and it would be interesting to explore the effects of these drugs on AMA. The reduction of sEV secretion with a drug targeting neutral sphingomyelinase 2 (nSMase2) also decreased Aβ plaque load in the mouse brain [3]. Similarly, senescent VSMCs showed increased nSMase2 expression and the use of inhibitors and siRNA targeting of nSMase2 decreased medin deposition and aggregation *in vitro*, highlighting this pathway as another potential target for intervention in the vasculature.

The pathological effects of medin accumulation in the vasculature, as well as the forms of medin responsible for inducing damage, remain poorly understood. Studies have shown that small medin aggregates can induce endothelial cell dysfunction and inflammation while accumulation of fibrillar amyloid species can contribute to weakening and stiffening of the vessel wall [6]. These effects may be particularly relevant to the cerebrovasculature as a recent study has shown that medin accumulates in the cerebral vessels with age [7] and may enhance Aβ formation, leading to vascular stiffening in the brain and increased CAA burden [8]. These data suggest that medin may represent a new therapeutic target for AD and CAA, to maintain a functioning and healthy cerebral environment during ageing. Further work is now required to understand the relationships between cellular ageing pathways, different forms of amyloidosis and potentially other ageing pathologies with shared mechanisms, such as vascular calcification, that often occur concomitantly within the aged ECM.
